# The Pheochromocytoma/Paraganglioma syndrome: an overview on mechanisms, diagnosis and management

**DOI:** 10.1590/S1677-5538.IBJU.2023.0038

**Published:** 2023-04-10

**Authors:** José Viana Lima, Claudio Elias Kater

**Affiliations:** 1 Divisão de Endocrinologia e Metabolismo Faculdade de Medicina da Santa Casa São Paulo SP Brasil Divisão de Endocrinologia e Metabolismo, Faculdade de Medicina da Santa Casa de São Paulo, São Paulo, SP, Brasil;; 2 Departamento de Medicina Escola Paulista de Medicina Universidade Federal de São Paulo São Paulo SP Brasil Unidade de Adrenal e Hipertensão, Divisão de Endocrinologia e Metabolismo, Departamento de Medicina, Escola Paulista de Medicina, Universidade Federal de São Paulo (EPM/UNIFESP), São Paulo, SP, Brasil

**Keywords:** Pheochromocytoma, Paraganglioma, Metanephrine

## Abstract

Pheochromocytomas/paragangliomas (PPGL) are rare, metastatic, and potentially fatal neuroendocrine tumors, often neglected because they present symptoms similar to other prevailing clinical conditions such panic syndrome, thyrotoxicosis, anxiety, hypoglycemia, etc., delaying diagnosis and treatment. The rate of diagnosis of PPGL has been increasing with the improvement in the measurement of catecholamine metabolites and the expanding availability of imaging procedures. Its essential genetic nature has been extensively investigated, comprising more than 20 genes currently related to PPGL and more new genes will probably be revealed. This overview will shed some light on the clinical, laboratory, topographical, genetic diagnosis, and management of PPGL.

## INTRODUCTION

Pheochromocytomas/paragangliomas (PPGL) are rare neuroendocrine tumors capable of producing, storing, and secreting catecholamines and other substances, such as VIP, PTH- and calcitonin-related peptides, opioids, CRH, ACTH, histamine, chromogranin, interleukin-6, etc (1-3).

PPGL is a serious, potentially metastatic, and fatal disease that often goes unnoticed by unexperienced doctors. Approximately 85-90% of PPGL are localized in the adrenals and 10-15% are extra-adrenal, being called paragangliomas (PGL); the latter may be found from the base of the skull to the testicles but are mostly found within the abdomen (4-7).

In this mini-review article we survey on clinical, laboratory, topographical, genetic, and therapeutic aspects of PPGL, a condition that has been showing an increase in incidence with the improvement of methods to measure catecholamine metabolites and imaging techniques.

## EPIDEMIOLOGY

The prevalence of PPGL among the hypertensive population is 1:500-1,000, but 75% of the cases are diagnosed *postmortem,* and in 55% of them PPGL directly contributed to death. In autopsy studies, the prevalence of PPGL ranges from 250 to 1,300 cases per million. Thus, clinical suspicion of PPGL still draws little attention (5, 6, 8).

The incidence of PPGL has been increasing over time, despite a fall in the number of necropsies, and this is due to the increase demand in the number of imaging exams and improved methods for measuring catecholamine metabolites (6).

## CLINICAL PICTURE AND INVESTIGATION

The symptomatology of patients with PPGL is variable. Systemic arterial hypertension (SAH) is the most frequent clinical manifestation of the disease, being present in 90% of cases. However, paroxysms (headache, palpitation, and sweating) are the most characteristic findings, resulting from release of catecholamines by the tumor and consequent stimulation of adrenergic receptors. They are often accompanied by increased blood pressure, tremor, pallor, chest or abdominal pain, and less commonly, facial flushing. Paroxysms do not occur in all patients. In some series, one or more components of the classical triad were present in more than 90% of patients. (4, 7-11)

The frequency of paroxysms is unpredictable and varies from 30 times a day to a single episode every 2-3 months. Near 75% of patients have one or more spells per week. Duration ranges from a few minutes (usually 15 to 60 min.) to days. They may arise spontaneously or be precipitated by activities that compress the tumor or elicit an increase in catecholamine secretion, such as exercises, pressure on the abdomen, urination, defecation, the act of smoking, and drugs like beta-blockers, anesthetic agents, radiologic contrasts, glucagon, metoclopramide, and tricyclic antidepressants (1-3, 6-13).

There are clinical scores based on signs and symptoms that have high diagnostic predictability. Among the signs and symptoms are hyperhidrosis, palpitation, pallor, tremor, nausea, heart rate >85 bpm plus body mass index (BMI) (14).

SAH may be paroxysmal, but more commonly are persistent (in ~60% of cases). It tends to be severe and/or refractory to antihypertensive medications and present with ample fluctuations. Sudden elevation of blood pressure (associated or not with other symptoms) may occur during abdominal manipulation, labor, intubation, anesthetic induction, surgery, or other invasive procedures. Norepinephrine (NE)-secreting tumors are usually associated with constant SAH, whereas those that secrete substantial amounts of epinephrine (E) in addition to NE are associated with episodic SAH. Conversely, when tumors secrete solely E, they provoke hypotension instead of hypertension; in this situation, the clinical feature may be of a cardiogenic shock. Orthostatic hypotension may be present in 40% of patients (12-14).

Cardiac abnormalities such as left ventricular hypertrophy occur quite commonly in patients with SAH, and myocarditis or dilated cardiomyopathy may result from circulating excess catecholamines. Palpitations and arrhythmias are common and occasionally fatal (12, 15).

Pre-diabetes is present in 50% of cases and diabetes mellitus (DM) in 10-20%. They are secondary to suppression of insulin secretion and increased hepatic glucose output, induced by excess catecholamines. Hypercalcemia may also occur due to concomitance of hyperparathyroidism or tumor production of PTH-related protein (PTHrp).

Atypical manifestations such as ACTH-dependent Cushing’s syndrome, acute abdomen, cardiovascular (shock, myocarditis, cardiac arrhythmias, acute pulmonary edema, heart failure, Takotsubo syndrome) and neurological events (altered mental status, seizures, stroke, and focal neurological manifestations), weight loss, fever of indeterminate origin, aqueous diarrhea, or constipation simulating pseudo-obstruction and paralytic ileus may also be found. Fever of mild to severe intensity (reaching up to 41^0^C) is not uncommon and has been attributed to IL-6 secretion (11-13).

## INVESTIGATION

Candidate subjects for a PPGL screening are: 1) young hypertensive patients under 30 years of age; 2) hypertensive patients refractory to treatment with 3 classes of antihypertensive drugs in effective doses; 3) hypertensive patients with paroxysms (headache, palpitation and sweating), seizures, unexplained shock, mucous neuromas, orthostatic hypotension, weight loss, presence of type I neurofibromatosis, family history of PPGL, medullary thyroid carcinoma, von Hippel-Lindau syndrome and familial PGL syndrome; 4) adrenal incidentalomas, especially in cases where pre-contrast attenuation values on computed tomography (CT) are ≥10 HU (Hounsfield units) and contrast washout <60%; 5) marked blood pressure lability; 6) episodes of shock or severe blood pressure responses during anesthesia induction, surgeries, invasive procedures, labor and use of β-blockers; 7) Takotsubo syndrome; 8) new-onset diabetes mellitus in a young lean hypertensive patient (12, 14).

## GENETICS

Approximately 25% of PPGL are genetic, and 50% of such patients have a pathogenic germline variant (PV). The following genes have already been associated with PPGL: ATM, DLST, EGLN1, EGLN2, FH, EPAS1 (HIF2A), HRAS, KIF1B, MAX, MDH2, MEN1, MERTK, MET, NF1, RET, SLC25A11, SDHA, SDHAF2, SDHB, SDHC, SDHD, TMEM127, TP53 and VHL (10-13, 16).

Hereditary PPGL are classified according to their transcription signature and are divided into three clusters as shown in Table-1.

**Table 1:** Transcriptional signature characteristics of hereditary PPGL.

Transcriptional signature		
Cluster 1 group (10-15%)	Cluster 2 group (50-60%)	Cluster 3 group (5-10%)
Cellular response to hypoxia	Proteins that activate kinase signaling	Via Wnt
Extra-adrenal syndrome + von Hippel-Lindau	Adrenal	Adrenal + Extra-adrenal
Germline / Somatic	Germline / Somatic	Somatic
Normetanephrine / 3-Methoxytyramine (3-MT)	Normetanephrine + metanephrine or metanephrine only	Normetanephrine metanephrine / Chromogranin A
SDHD, SDHC, SDHB, SDHA, SDHA2F, VHL, HIF, FH, EGLN1 (PHD2), EGLN2 (PHD1), KIF 1β, EPAS1/2 (HIF2A), MDH2	RET, NF1, MAX, TMEM127, HRAS	CSDE1, MAML3

Next (and in Table-2) we describe briefly the main syndromic features that are associated with specific PPGL syndromes:

**Table 2:** Main syndromic features associated with specific hereditary PPGL.

Gene	Syndrome	Tumor location	Rate of PPGL metastases	Association with other tumors
**NF1**	Neurofibromatosis type 1	Mostly adrenal (bilateral)	12%	Neurofibromas, malignant tumors of the peripheral nerve sheath, optic gliomas and leukemias
**RET**	Multiple endocrine neoplasia type 2	Adrenal (bilateral)	<5%	Medullary thyroid carcinoma, parathyroid adenomas/ hyperplasia
**VHL**	von Hippel Lindau	Mostly adrenal (bilateral)	5-8 %	Renal clear cell (RCC) carcinoma, neuroendocrine tumors of the pancreas (mostly non-functioning), CNS hemangioblastomas, endolymphatic sac tumors, pituitary adenomas
**SDHA**	Hereditary PGL syndrome	Any	30-60%	RCC carcinoma, gastro-intestinal stromal tumors (GIST) and pituitary adenomas
**SDHB**	Hereditary PGL syndrome	Any, mostly extra-adrenal	35-75%	RCC carcinoma, GIST and pituitary adenomas
**SDHC**	Hereditary PGL syndrome	Head and neck, can be thoracic	Low	RCC carcinoma, GIST and pituitary adenomas
**SDHD**	Hereditary PGL syndrome	Any, mostly head and neck	15-29%	RCC carcinoma, GIST and pituitary adenomas
**SDHAF 2 (SDH5)**	Hereditary PGL syndrome	Head and neck (multifocal)	Not Known	RCC carcinoma, GIST and pituitary adenoma
**TMEM 127**	Familial PGL syndrome	Any, mostly adrenal	Low	RCC carcinoma
**MAX**	Familial PGL syndrome	Mostly adrenal (bilateral)	Intermediate to high	Pituitary adenomas
**EPAS1**	Familial PGL syndrome, polycythemia	Any	Unknown	Somatostatinoma
**FH**	Hereditary leiomatosis, RCC carcinoma	Any	Possibly high	Cutaneous and uterine leiomyomas, renal papillary carcinoma
**MDH2**		Any	Unknown	

### von Hippel-Lindau (VHL) Syndrome

PPGL occurs in 10 to 30% of patients with VHL. The VHL syndrome is classified as: type 1, in which PPGL does not manifest, and type 2, which is subdivided into 3 subtypes: 2A (encompassing PPGL plus retinal and CNS hemangioblastomas, and low risk for renal carcinoma), 2B (PPGL plus retinal and CNS hemangioblastomas and kidney and pancreatic tumors), and 2C (PPGL only).

PV occur in the *VHL* gene, which is a tumor suppressor located on chromosome 3p25, responsible for regulating hypoxia-induced genes by ubiquitination and subsequent degradation of HIF2α. VHL disease has a penetration >90% at 65 years of age and *missense* PV are likely associated with the development of PPGL, whereas truncated or large variants are associated with the presence of hemangioblastomas and renal cell carcinoma (17-22).

### Paragangliomas

PV of succinate dehydrogenase (SDH) subunits D, B, C, A, and A2F are associated with PGL. These subunits are related to signals responsive to oxygen level so that PV in the respective genes would lead to a chronic state of hypoxia and, therefore, cell proliferation. PGL are classified as follows:

**PGL1:** results from PV in SDHD, located on chromosome 11q23, with a maternal *imprint* mechanism, which results in the PV almost always being transmitted by the father and a PV frequency of 3 to 5%, penetrance of 31 to 50% and frequency of metastases less than 5%; these PGL are usually located in the head, neck, and adrenals bilaterally, and may or may not be functioning. In 75% of cases, the disease manifests around the age of 40 years. Renal carcinomas are found in 8% and pituitary adenomas have been reported in a few cases.

**PGL2:** results from PV of the *SDHA2F* gene. Initially described in 2009, this PV is rarely found in PGL. Located on chromosome 11q13 and, as in cases that present PV in *SDHD*, transmission is also by maternal *imprint* and almost always results from paternal transmission. PGL usually appear around 22 years of age and are often multifocal, although non metastatic.

**PGL3:** results from PV of the *SDHC* gene, located on chromosome 1q21, with autosomal dominant transmission, and PV frequency below 0.1%, unknown penetrance and indeterminate frequency of metastases; tumors in PGL3 localize in the head and neck and are not functioning.

**PGL4:** results from PV in the *SDHB* gene, located on chromosome 1p36.3, with autosomal dominant inheritance, and frequency PV ranging from 2 to 7%, penetrance of 50 to 70% and frequency of metastases from 34 to 70%; these PGL are usually located in the thorax, abdomen and adrenal bilaterally and are always functioning. Renal carcinomas occur in 14% and GIST in 2% of cases.

**PGL5:** results from PV of the *SDHA* gene that rarely cause PGL; corresponds to 3% of cases and has low penetrance. GIST and pituitary adenomas may be present. (10-13, 23, 24).

### Neurofibromatosis (NF)

PPGL may be associated with type 1 NF, whose diagnosis is clinical and generally does not pose diagnostic problems. The *NF-1* gene localize on chromosome 17q11.2 and is responsible for encoding a protein called neurofibromine; its inheritance is autosomal dominant. In NF-1, PV inactivate the gene and occur in 1 to 5% of the cases, when PPGL is not accompanied by hypertension and in up to 50% of those with hypertension. PPGL associated to PV in NF-1 is similar to sporadic ones, occurring in older patients; less frequently they are bilateral and extra-adrenal. PPGL was present in 3 to 13% of individuals who underwent autopsy (10-13, 23, 24-26).

### Multiple Endocrine Neoplasia (MEN)

In MEN 2A (medullary thyroid carcinoma [MTC], PPGL, and primary hyperparathyroidism) and 2B (MTC, PPGL, and mucous neuromas/intestinal ganglioneuromas and marfanoid habit), PPGL may be present in 50% of cases. PV in the *RET* proto-oncogene (*Rearranged During Transfection*, localized on chromosome 10q11.2) is of *missense* germline. This gene encodes a tyrosine-kinase receptor that is expressed in various tissues derived from the neural crest, including the CNS and peripheral nervous system, and neuroendocrine tissues. *RET* PVs causing *MEN* 2A are mostly located in codons 609, 611, 618, 620 (exon 10) and 634 (exon 11). Although the most affected exons are 10, 11 and 16, PV in exons 13, 14 and 15 have also been reported. In MEN 2A codon 634 is the most affected. PV in codon 918 in exon 16 (methionine for threonine, M918T) are associated with 95% of cases of MEN 2B (27, 28).

#### TMEM127

The *TMEM127* gene, described by Dahia et al. in 2010, is positioned on chromosome 2q11; it is a tumor suppressor that, like the NF-1 gene, promotes gene inactivation (20). In a cohort of 103 samples, PV was present in 30% of cases and in 3% of apparently sporadic PPGL (23, 24).

## Laboratory Diagnosis

Laboratory diagnosis of PPGL is usually accomplished by measuring blood and urine metanephrines. The current gold standard is a plasma metanephrine (MN) measurement that achieves a sensitivity of 99% for sporadic and hereditary functioning PPGL and a specificity of 99% for hereditary (and 89% for sporadic), superior to any combination of tests. Normal plasma MN virtually excludes functioning PPGL. Preferably, plasma MN and/or urinary MN should be the tests of choice for the diagnosis of PPGL.

Chromogranin A (ChrA), an acid glycopeptide co-secreted by PPGL, can be measured during laboratory investigation; it has a diagnostic sensitivity of 83-86% and specificity of 76-98%. ChrA is not influenced by antihypertensive drugs and exhibits an increase in positive predictive value (PPV) when combined with plasma MN. ChrA may be elevated in cervical PGL that do not have elevated plasma and/or urinary MN, thus functioning as a tumor marker in this situation. However, ChrA may be increased in the following conditions: renal failure (creatinine clearance <80mL/min), use of proton pump inhibitors, liver failure, and atrophic gastritis. Also, ChrA has low specificity since other neuroendocrine tumors (NET) can also produce it.

When plasma MN concentration is only 2-4 times above normal values, a clonidine test can be performed using plasma MN measurements at baseline and 3 hours after oral administration of 0.3 mg clonidine. Suppression below 40% suggests PPGL. Vanillylmandelic acid, urinary and plasma catecholamines, and the classic glucagon and clonidine tests using plasma catecholamine measurements are no longer used (7, 8, 12, 13, 29-33).

**Figure 1:**
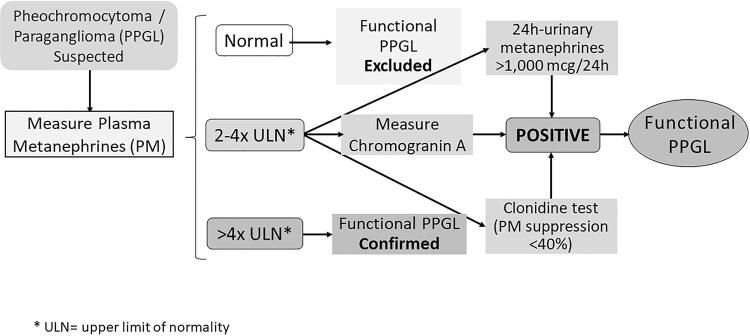
Flowchart for the diagnosis of functioning PPGL.

In Figure-1, we described a laboratory flowchart for the diagnosis of functioning PPGL.

## Imaging / Localization Diagnosis

Localization of PPGL can be achieved by the following procedures (all employing specific protocols for the adrenals): (1) magnetic resonance imaging (MRI) of the upper abdomen or whole body (when PGL is suspected), (2) computed tomography (CT) of the upper abdomen, (3) full body scintigraphy with ^123^I/^131^I-mIBG (metaiodo-benzylguanidine), (4) PET-CT with^18^FDG, and (5) PET-CT with ^68^Ga DOTATATE, DOTATOC or DOTANOC.

**Figure 2:**
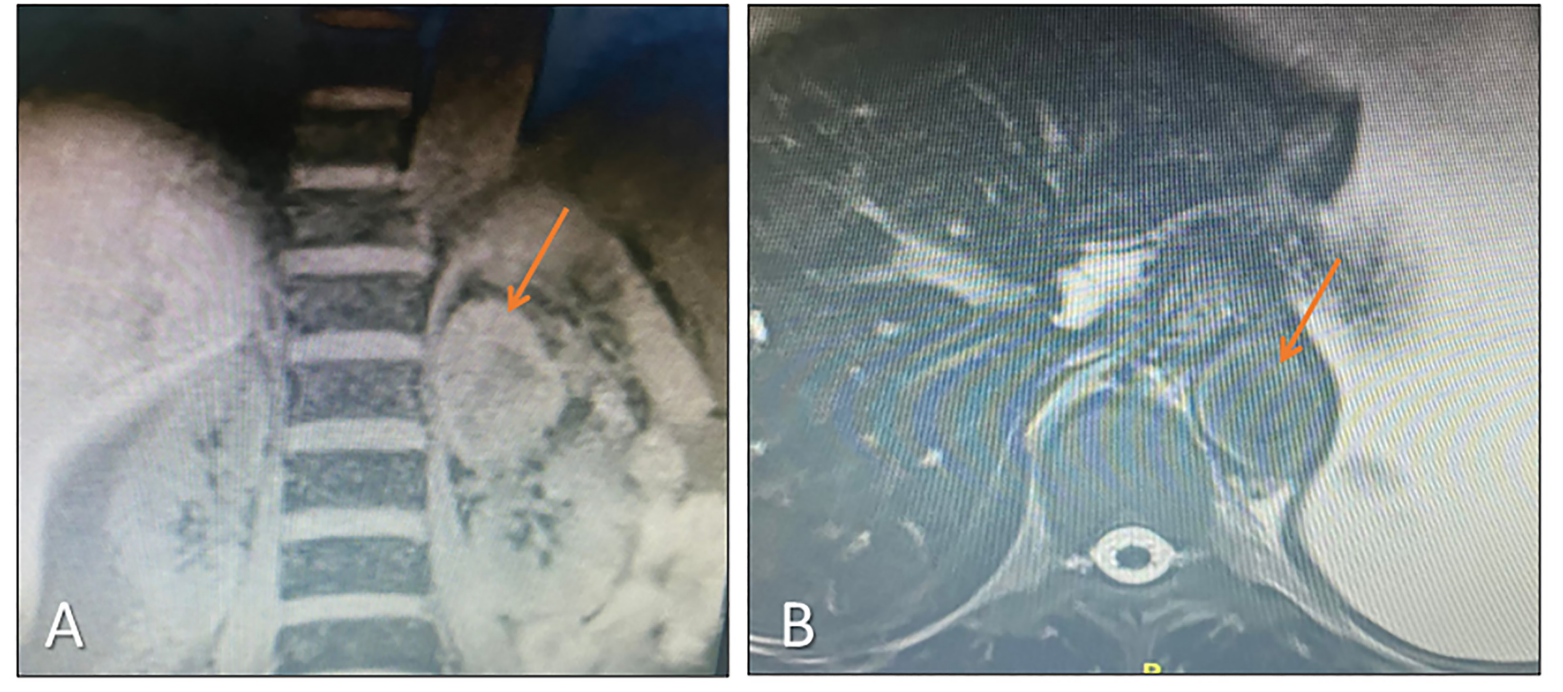
Left adrenal pheochromocytoma in a 63 yo patient. A: 6.3 cm lesion showing a cleavage plan with necrosis (MRI, coronal section). B: Chemical shift does not show loss of signal in the out-of-phase sequence (MRI, axial section).

Use of MRI for the diagnosis of PPGL has the following advantages: (1) high sensitivity (93-100%) in detecting adrenal disease, (2) presence of a “hypersignal” in T2 sequence compared to the liver, in at least 75% of PPGL, (3) better sensitivity to localize intracardiac PGL, (4) possibility of visualization and confirmation of bone metastases suggested by mIBG scintigraphy, and (5) can be performed in pregnant women (second trimester on) (without contrast) and in children and carriers of germline variants, since there is no exposure to ionizing radiation. In Figure-2, we described the MRI with sporadic pheochromocytoma on the left adrenal with some typical features.

CT has a sensitivity of 93-100%, but low specificity (70%). Sensitivity is lower for small adrenal PPGL and for adrenal medullary hyperplasia. It is also less sensitive in the detection of PGL, small metastases and early recurrence of tumors in the adrenal surgical bed. CT is currently recommended as the first choice for topographic diagnosis of PPGL (11, 25, 30, 34, 36-38).

^131^I-mIBG scintigraphy has diagnostic sensitivity and specificity of 77-90% and 95-100%, respectively. When ^123^I is used instead, sensitivity reaches higher values: 83-100%, without loss of specificity. Its use should be considered in cases of adrenal Pheo that are suggestive of benignity. False negative results occur in 15% (approximately 60% of PGL are not avid for mIBG), and false positives can also occur, since 50% of normal adrenals have physiological uptake. The following are indications for pre-surgery mIBG: diagnostic confirmation, inconclusive biochemical results, familial disease, extra-adrenal tumors, and the possibility of treatment with therapeutic mIBG in metastatic PPGL. Post-surgical mIBG are indicated to search for disease recurrence and metastases (1, 5, 7-9).

**Figure 3:**
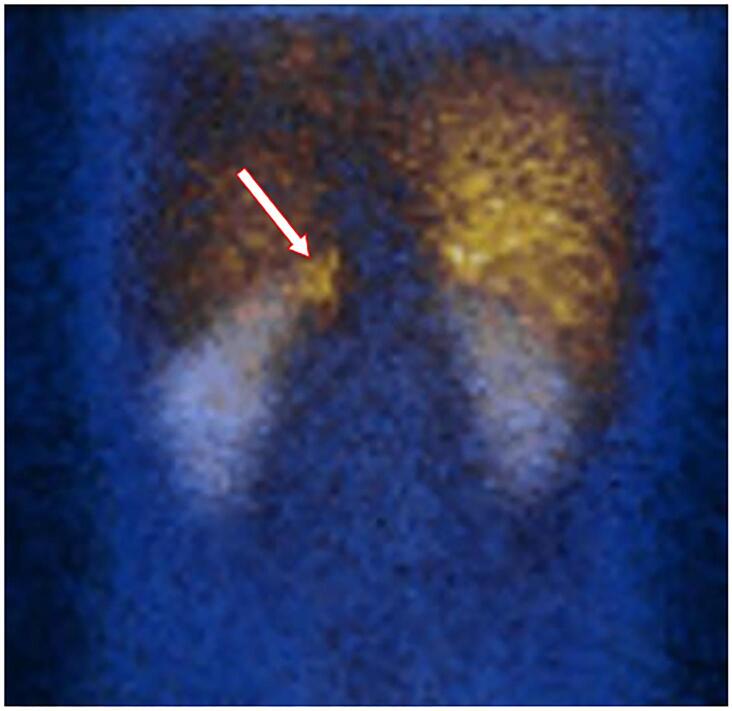
Coronal 123I-MIBG scintigraphy (posterior image) showing increased focal radiotracer uptake in the left adrenal (arrow) in a young male with a pheochromocytoma and MEN2B.

^18^FDG PET-CT is recommended for aggressive metastatic PPGL, lesions greater than 8 cm and those with PV in the *SDHB* gene. Sensitivity ranges from 74-100%. In Figure-3, we described a Coronal ^123^I-MIBG scintigraphy showing increased focal radiotracer uptake in the left adrenal in a young male with a pheochromocytoma and MEN2B.

^68^Ga PET-CT DOTATATE, DOTATOC or DOTANOC have high sensitivity and specificity for neuroendocrine tumors as well as for tumor dedifferentiation; its recommendations parallel those of ^18^FDG PET-CT (11,25,32,34). The histological concept of malignancy in PPGL is rather complex, since histological features of malignancy can be identified in “benign” PPGL, and histological absence of malignancy may be found in “malignant” tumors. Thus, malignancy is defined when there is evidence of distant metastasis; however, large Pheo (>8 cm), PGL with increased production of dopamine/methoxy-tyramine (dopamine metabolite) also suggest “malignancy”. Since 2018, the World Health Organization (WHO) has recommended the terms metastatic and non-metastatic, instead of malignant and benign PPGL (7,11, 12, 25, 32, 35-37).

The most used histological classification to aid in establishing malignancy potential is the PASS score (Pheochromocytoma of the Adrenal Gland Scaled Score) which considers the following items (Table-3):

**Table 3:** The “Pheochromocytoma of the Adrenal Gland Scaled Score” or PASS score.

ITEMS CONSIDERED	PASS Score
Diffuse growth pattern or in "large nests"	2
Focal or diffuse necrosis	2
High cellularity	2
Cellular monotony	2
Tumor with spiculated cells	2
Mitotic index >3/10 large increase field	2
Atypical mitoses	2
Vascular invasion	1
Capsular invasion	1
Extension to adjacent adipose tissue	1
Intense nuclear pleomorphism	1
Nuclear hypercromasia	1

Non-metastatic PPGL have a score ≤3 and those potentially more aggressive ≥4 points. To date, there is no stratification model that combines histological and genetic data.

In the GAPP system, histological classification is based on the scoring system composed of 6 parameters: histologic pattern, cellularity, necrosis, capsular/ vascular invasion, in association with immunohistochemistry (Ki-67) and hormonal secretion (production of noradrenaline or normetanephrine or associated with dopamine/methoxy-tyramine has 1 point), totaling 10 points. According to the GAPP system, patients are classified into 3 classes: 1) well differentiated: 0-2 points; 2) moderately differentiated: 3-6 points; and 3) poorly differentiated: 7-10 points (11,12,35).

Tumor immunohistochemistry for the succinate dehydrogenases, especially the investigation of *SDHB* is indicated, as the loss of its expression suggests a germline PV in the *SDHB* gene and implies greater aggressiveness; this analysis is part of COOPS (Composite Pheochromocytoma/Paraganglioma Prognostic Score) system, in which necrosis (focal or confluent), loss of S100 expression, vascular invasion, loss of *SDHB* expression and size greater than 7 cm are evaluated. Scores greater ≥3 have a higher risk of metastasis (12, 35).

The 8^th^ edition of the AJCC (American Joint Committee on Cancer) staging system includes a special chapter for PPGL, but not for parasympathetic PGL, as metastatic behavior is less than 5%. Pheo smaller than 5 cm in their longest axis and without vascular invasion are classified as T1; those ≥5 cm or sympathetic PGL of any size and without extra-adrenal invasion are classified as T2. PPGL of any size with invasion of surrounding tissues such as liver, pancreas, spleen and kidneys are classified as T3. Regarding lymph node involvement: Nx (without knowledge of involvement), N0 (without involvement of lymph nodes) and N1 (with involvement of regional lymph nodes). Regarding distant metastases, M0 (no distant metastases), M1a (distant metastases to bone only), M1b (distant metastases to distant lymph nodes/liver or lung) and M1c (distant metastases to bone and multiple other organs).

Classification is as follows: Stage I: T1N0M0 / Stage II: T2N0M0 / Stage III: T1N1M0, T2N1M0, T3 any N and M0 / Stage IV: any T, any N and M1 (12, 35).

## Clinical treatment

Treatment of PPGL is surgical whenever possible since there is a possibility of reversal of SAH. In addition, complications of an untreated PPGL can be fatal and there is a chance of metastases in 15-17% of cases.

Preoperative clinical therapy for a minimum of 7-30 days (15 days, on average) is mandatory, aiming to prevent an intraoperative hypertensive crises and cardiac arrhythmias, and to avoid hypotension after tumor removal. The best drugs for this purpose are α-blockers, such as prazosin, doxazosin, and terazosin; phenoxybenzamine has been less accepted in Brazil, as it has a longer biological half-life (and should be withdrawn 48h preoperatively, leaving the patient a period of 2 days rather unprotected) and may produce reflex tachycardia after its withdrawal.

Prazosin and doxazosin are the most widely used drugs, in doses ranging from 1 to 16-20 mg per day. On average, 12 mg prazosin and 10 mg of doxazosin warrant good blood pressure control and prevention of paroxysms. Additionally, calcium channel blockers (amlodipine, diltiazen, verapamil and nifedipine) and angiotensin-converting enzyme (ACE) inhibitors may also be used. The use of β-blockers should be kept for when tachycardia and tachyarrhythmias are present, but always after effective control of hypertension with α-blockade; on average, β-blockers may be used after 3 days of the introduction of α-blockade.

α-Methyl-paratyrosine blocks the synthesis of catecholamines by inhibiting tyrosine hydroxylase, a key enzyme in the hormonal synthesis process; it can reduce catecholamine excretion by 35-80%. In general, it is recommended to treat SAH in patients with unresectable tumors or in those with metastases and in the preoperative period when there is no effective control with α-adrenergic blockers. Initial dose is 250mg 4x per day, a dose that can be adjusted every 3-4 days according to blood pressure response and possible side effects (sedation, psychiatric disorders, extrapyramidal symptoms, urolithiasis). The largest recommended dose is 4g/day (2, 8, 12, 15, 32).

### SURGICAL TREATMENT

Only experienced surgeons and anesthesiologists should be responsible for the PPGL surgical procedure. The laparoscopic approach is preferred for tumor access, except for cases of suspected metastases and tumor size larger than 7 cm, conditions in which the classic open access is mandatory. Ideally, the entire immediate postoperative (post-op) period should be done in an intensive care unit (ICU), because even with adequate preparation there is a risk of arrhythmias and blood pressure instability, with the possibility of hypertensive crises and hypotension in the post-op period. There is still also a risk of hypoglycemia in the post-op, and installation of a 10% IV glucose solution is recommended for a period of 48h, with capillary glucose controls. The patient may remain hypertensive for a period of 2 weeks, after which a new 24h-plasma and/or urinary MN measurement is recommended.

For PPGL patients with metastases, the target is to achieve tumor reduction and to control hypertension. Large PPGL can be reduced through surgery to obtain symptom relief and control of blood pressure levels; however, rarely will this surgery be curative, as there are often distant metastases, especially in bones (70%). Exceptionally, when metastases are restricted to the liver but are not surgically removable, transplantation will be an option. Tumor reduction can also be achieved by other interventional techniques such as transcatheter selective embolization or chemoembolization.

Thermal perfusion of the liver with cytotoxic drugs is used in some centers in cases of hepatic metastases.

Alternatives for surgical resection in cases of metastatic PPGL include radiotherapy (effective for bone pain), cryoablation, and radiofrequency thermal ablation (2, 8, 12, 15, 32).

## Treatment with 131I-mIBG

The use of radiolabeled mIBG in metastatic PPGL therapy should be considered, as mIBG may cross the cell membrane and be stored in cytoplasmic granules via VMS transporters (VMAT1 and 2). Since 1984, several patients with PPGL have been treated using different therapeutic protocols. Such patients are selected by demonstrating significant uptake of the radioisotope during a scintigraphy with ^123^I/^131^I mIBG.

The only impediment to this treatment is the total dose of radiation delivered to vital organs, such as bone marrow. Approximately 60% of metastases are avid for ^131^I-mIBG. Recently, quantitative determination of VMAT 1 and 2 expression in surgical specimens proved useful in selecting patients suitable for treatment with ^131^I-mIBG (12, 36, 37).

A review of 116 patients treated with 100 to 300 mCi of ^131^I-mIBG per session (mean of 3 doses at intervals of 3-14 months) showed tumor shrinkage in 30% of patients, disease stabilization in 57% and progression in 13%. A positive hormonal response ranged from 15 to 45%. (12, 24, 36-38).

In general, patients with limited disease have an increased chance of tumor response. Similarly, soft tissue metastases respond better than bone metastases. Hormonal and symptomatic responses to ^131^I-mIBG are independent of tumor size response (36-38).

Major side effects include transient leukopenia and thrombocytopenia. Myelosuppression, infections, and liver failure are rare occurrences in patients with spread liver metastases (11, 12, 36-38).

## Treatment with radioactive somatostatin analogues

Due to the expression of somatostatin receptors in metastatic PPGL, the use of radiopharmaceuticals (RP) based on somatostatin analogues has been tested.

Several RP with different physical properties is employed, including octreotide-^111^In-DOTA /pentreotide-^111^In-DOTA, octreotide-^90^Y-DOTA-, octreotate-^177^Lu-DOTA, plus lanreotide-^111^In-DOTA and lanreotide-^90^Y-DOTA (25, 28, 32).

Patients who will benefit from treatment are those who have an increased tumor uptake on scintigraphy (currently ^68^Ga PET-CT with DOTATATE, DOTATOC or DOTANOC).

Stabilization or decrease in hormonal secretion and tumor growth have been reported in 20-25% of cases. Main side effects include leukopenia and thrombocytopenia.

Treatment with unlabeled octreotide is generally unsuccessful and only in some patients a transient response was observed because they express a low density of subtype 2 somatostatin receptors (SST2) (7, 11, 12).

## Chemotherapy

Chemotherapy (QT) is an option when the tumor is inoperable and/or there is extensive residual disease. The combination of cyclophosphamide, vincristine and dacarbazine (CVD) may provide partial remission and transient symptomatic relief in up to 50% of patients with metastatic PPGL, although short-lived (1, 7, 9, 11, 12, 36, 37).

Other QT options are etoposide and cisplatin, anthracycline plus CVD and arabinoside cytokine. Some authors suggest a combination of lomustine and 5-fluorouracil or capecitabine for tumors with slow progression, whereas for rapidly progressive tumors, the best option would be the association of etoposide with a drug based and platinum (7, 9, 11, 12, 36, 37).

## New and emerging therapies

New antineoplastic therapies are being tested in patients with metastatic PPGL. The combination of temozolomide and thalidomide provided biochemical and radiological responses in 40 and 33% of the cases, respectively; however, lymphopenia accompanied by opportunistic infections occurred in most patients.

Other therapeutic options include 17-alilamine protein inhibitors (17-demethoxy-gel-danamycin), mTOR inhibitors (everolimus), tyrosine-kinase inhibitors with anti-VEGF activity, antiangiogenic factors, gene therapy, etc. Lutetium-octreotate has relatively few side effects and can complement the effect of 131I-mIBG for small lesions or micro-metastases (7, 9, 11, 12, 36, 37).

## Follow-up

Patients with PPGL should undergo annual reevaluations by measuring urinary or plasma MN and chromogranin A. Follow-up is for life. When there is clinical and laboratory recurrence, with no radiological evidence, a new full body scintigraphy should be performed with ^123^I/^131^I-mIBG, or^68^Ga PET-CT with DOTATE, DOTATOC or DOTANOC or ^18^FDG-PET-CT (11-13).

There are specific follow-up protocols for some genetic syndromes such as MEN2A and 2B, Von Hippel Lindau syndrome and familial paraganglioma syndrome (10, 12, 22, 25-28).

## CONCLUSIONS

The PPGL syndrome is a rare condition, but the improvement of catecholamine metabolite assays and topographic/functional location procedures, have helped to demonstrate its actual higher incidence. Recognizing and treating hypertensive patients with PPGL is extremely important, avoiding serious cardiovascular complications, metastases and death.

The importance of genetics in PPGL is essential nowadays, with more PPGL-related genes being discovered, which allows better treatment strategies, monitoring of genetic syndromes related to PPGL, and familial counselling.

The treatment and follow-up of PPGL should be carried out by a multidisciplinary team with experience in this disease, composed of endocrinologists, radiologists, radio-interventional physicians, nuclear physicians, anesthesiologists, geneticists, urologists/oncological surgeons, head and neck surgeons/neurosurgeons (for head and neck PGL), thoracic surgeons (for thoracic PGL), intensive care physicians, pathologists, clinical oncologists, radiotherapist physicians and psychologists.

## References

[1] Chrisoulidou A, Kaltsas G, Ilias I, Grossman AB (2007). The diagnosis and management of malignant phaeochromocytoma and paraganglioma. Endocr Relat Cancer.

[2] Karagiannis A, Mikhailidis DP, Athyros VG, Harsoulis F (2007). Pheochromocytoma: an update on genetics and management. Endocr Relat Cancer.

[3] Erlic Z, Rybicki L, Peczkowska M, Golcher H, Kann PH, Brauckhoff M (2009). Clinical predictors and algorithm for the genetic diagnosis of pheochromocytoma patients. Clin Cancer Res.

[4] Ilias I, Pacak K (2004). Current approaches and recommended algorithm for the diagnostic localization of pheochromocytoma. J Clin Endocrinol Metab.

[5] Sutton MG, Sheps SG, Lie JT (1981). Prevalence of clinically unsuspected pheochromocytoma. Review of a 50-year autopsy series. Mayo Clin Proc.

[6] Al Subhi AR, Boyle V, Elston MS (2022). Systematic Review: Incidence of Pheochromocytoma and Paraganglioma Over 70 Years. J Endocr Soc.

[7] Neumann HPH, Young WF, Eng C (2019). Pheochromocytoma and Paraganglioma. N Engl J Med.

[8] Bravo EL, Tagle R (2003). Pheochromocytoma: state-of-the-art and future prospects. Endocr Rev.

[9] Adler JT, Meyer-Rochow GY, Chen H, Benn DE, Robinson BG, Sippel RS (2008). Pheochromocytoma: current approaches and future directions. Oncologist.

[10] Benn DE, Gimenez-Roqueplo AP, Reilly JR, Bertherat J, Burgess J, Byth K (2006). Clinical presentation and penetrance of pheochromocytoma/paraganglioma syndromes. J Clin Endocrinol Metab.

[11] Lenders JW, Duh QY, Eisenhofer G, Gimenez-Roqueplo AP, Grebe SK, Murad MH, N (2014). Pheochromocytoma and paraganglioma: an endocrine society clinical practice guideline. J Clin Endocrinol Metab.

[12] Garcia-Carbonero R, Matute Teresa F, Mercader-Cidoncha E, Mitjavila-Casanovas M, Robledo M, Tena I (2021). Multidisciplinary practice guidelines for the diagnosis, genetic counseling and treatment of pheochromocytomas and paragangliomas. Clin Transl Oncol.

[13] Jiang J, Zhang J, Pang Y, Bechmann N, Li M, Monteagudo M (2020). Sino-European Differences in the Genetic Landscape and Clinical Presentation of Pheochromocytoma and Paraganglioma. J Clin Endocrinol Metab.

[14] Geroula A, Deutschbein T, Langton K, Masjkur J, Pamporaki C, Peitzsch M (2019). Pheochromocytoma and paraganglioma: clinical feature-based disease probability in relation to catecholamine biochemistry and reason for disease suspicion. Eur J Endocrinol.

[15] Bruynzeel H, Feelders RA, Groenland TH, van den Meiracker AH, van Eijck CH, Lange JF (2010). Risk Factors for Hemodynamic Instability during Surgery for Pheochromocytoma. J Clin Endocrinol Metab.

[16] Eisenhofer G, Lenders JW, Linehan WM, Walther MM, Goldstein DS, Keiser HR (1999). Plasma normetanephrine and metanephrine for detecting pheochromocytoma in von Hippel-Lindau disease and multiple endocrine neoplasia type 2. N Engl J Med.

[17] Friedrich CA (1999). Von Hippel-Lindau syndrome. A pleomorphic condition. Cancer.

[18] Sansó G, Rudaz MC, Levin G, Barontini M (2004). Familial isolated pheochromocytoma presenting a new mutation in the von Hippel-Lindau gene. Am J Hypertens.

[19] Arao T, Okada Y, Tanikawa T, Inatomi H, Shuin T, Fujihira T (2002). A case of von Hippel-Lindau disease with bilateral pheochromocytoma, renal cell carcinoma, pelvic tumor, spinal hemangioblastoma and primary hyperparathyroidism. Endocr J.

[20] Dahia PL, Consortium Familial Pheochromocytoma (2006). Transcription association of VHL and SDH mutations link hypoxia and oxidoreductase signals in pheochromocytomas. Ann N Y Acad Sci.

[21] Richards S, Aziz N, Bale S, Bick D, Das S, Gastier-Foster J (2015). Standards and guidelines for the interpretation of sequence variants: a joint consensus recommendation of the American College of Medical Genetics and Genomics and the Association for Molecular Pathology. Genet Med.

[22] Rednam SP, Erez A, Druker H, Janeway KA, Kamihara J, Kohlmann WK (2017). Von Hippel-Lindau and Hereditary Pheochromocytoma/Paraganglioma Syndromes: Clinical Features, Genetics, and Surveillance Recommendations in Childhood. Clin Cancer Res.

[23] Qin Y, Yao L, King EE, Buddavarapu K, Lenci RE, Chocron ES (2010). Germline mutations in TMEM127 confer susceptibility to pheochromocytoma. Nat Genet.

[24] Bausch B, Schiavi F, Ni Y, Welander J, Patocs A, Ngeow J (2017). Clinical Characterization of the Pheochromocytoma and Paraganglioma Susceptibility Genes SDHA, TMEM127, MAX, and SDHAF2 for Gene-Informed Prevention. JAMA Oncol.

[25] Nölting S, Bechmann N, Taieb D, Beuschlein F, Fassnacht M, Kroiss M (2022). Personalized Management of Pheochromocytoma and Paraganglioma. Endocr Rev.

[26] Yeh IT, Lenci RE, Qin Y, Buddavarapu K, Ligon AH, Leteurtre E (2008). A germline mutation of the KIF1B beta gene on 1p36 in a family with neural and nonneural tumors. Hum Genet.

[27] Wells SA, Asa SL, Dralle H, Elisei R, Evans DB, Gagel RF (2015). Revised American Thyroid Association guidelines for the management of medullary thyroid carcinoma. Thyroid.

[28] Thomas CM, Asa SL, Ezzat S, Sawka AM, Goldstein D (2019). Diagnosis and pathologic characteristics of medullary thyroid carcinoma-review of current guidelines. Curr Oncol.

[29] Eisenhofer G, Keiser H, Friberg P, Mezey E, Huynh TT, Hiremagalur B (1998). Plasma metanephrines are markers of pheochromocytoma produced by catechol-O-methyltransferase within tumors. J Clin Endocrinol Metab.

[30] Algeciras-Schimnich A, Preissner CM, Young WF, Singh RJ, Grebe SK (2008). Plasma chromogranin A or urine fractionated metanephrines follow-up testing improves the diagnostic accuracy of plasma fractionated metanephrines for pheochromocytoma. J Clin Endocrinol Metab.

[31] Boyle JG, Davidson DF, Perry CG, Connell JM (2007). Comparison of diagnostic accuracy of urinary free metanephrines, vanillyl mandelic Acid, and catecholamines and plasma catecholamines for diagnosis of pheochromocytoma. J Clin Endocrinol Metab.

[32] Antonio K, Valdez MMN, Mercado-Asis L, Taïeb D, Pacak K (2020). Pheochromocytoma/paraganglioma: recent updates in genetics, biochemistry, immunohistochemistry, metabolomics, imaging and therapeutic options. Gland Surg.

[33] Lenders JW, Pacak K, Walther MM, Linehan WM, Mannelli M, Friberg P (2002). Biochemical diagnosis of pheochromocytoma: which test is best?. JAMA.

[34] Chang CA, Pattison DA, Tothill RW, Kong G, Akhurst TJ, Hicks RJ (2016). (68)Ga-DOTATATE and (18)F-FDG PET/CT in Paraganglioma and Pheochromocytoma: utility, patterns and heterogeneity. Cancer Imaging.

[35] Mete O, Asa SL, Gill AJ, Kimura N, de Krijger RR, Tischler A (2022). Overview of the 2022 WHO Classification of Paragangliomas and Pheochromocytomas. Endocr Pathol.

[36] Kaltsas GA, Mukheyer JJ, Buton KE, Grossman AB (2003). Treatment of metastatic pheochromocytoma and paraganglioma with 131I-MIBG. The Endocrinologist.

[37] Scholz T, Eisenhofer G, Pacak K, Dralle H, Lehnert H (2007). Clinical review: Current treatment of malignant pheochromocytoma. J Clin Endocrinol Metab.

[38] Mozley PD, Kim CK, Mohsin J, Jatlow A, Gosfield E, Alavi A (1994). The efficacy of iodine-123-MIBG as a screening test for pheochromocytoma. J Nucl Med.

